# Supplementary material for: Conformational Dynamics of Mitochondrial Inorganic Pyrophosphatase hPPA2 and Its Changes Caused by Pathogenic Mutations
Source: Life (Basel). 2025 Jan 15;15(1):100. doi: 10.3390/life15010100 (PMC11766831; doi:10.3390/life15010100)
Supplement: Supplementary file 1 [file life-15-00100-s001.zip › life-3371619-supplementary.pdf]

Supplementary Materials

Table S1. Predicted effect of the pathogenic mutations on the hPPA2 fold stability calculated using Strum [24].

Residue in WT hPPA2	Patho-genic mutation	Calculated parameters for the pathogenic mutant variant		Aggregated parameters summed for all possible mutations		Conser-vation score/ grade*	Variations in PPases**
		ΔΔG, kcal/mol	Mutation score	ΔΔG, kcal/mol	Mutation score		
Ser61	Phe	0.29	1.33	9.66	32.0	-1.29/9	N, S
Met94	Val	-0.19	0.83	-2.95	16.5	-0.82/8	A, C, F, I, M, V, Y
Met106	Ile	0.58	1.79	8.83	30.5	-0.72/7	F, H, I, L, M, Q, S, V, Y
Arg127	Leu	1.99	7.32	25.75	75.6	-1.25/9	A, R, T
Thr148	Ser	0.76	2.14	9.61	32.1	-1.30/9	T
Pro167	Leu	-0.04	0.96	1.84	21.2	-1.29/9	P
Glu172	Lys	-0.16	0.85	-2.07	17.2	-1.05/9	D, E, Q
Trp202	Cys	-2.23	1.55	-2.97	35.9	-1.13/9	H, W, Y
Pro228	Leu	-0.35	1.75	-0.70	35.4	-0.87/8	D, E, F, G, H, K, P, R, S
His272	Tyr	0.91	1.31	0.21	25.6	-0.63/7	A, F, H, N, Q, S, Y
Gln294	Pro	-0.51	2.32	0.13	40.3	-0.004/5	A, C, E, F, H, K, L, N, Q, S, T

*The normalized conservation score is calculated using ConSurf server (https://consurf.tau.ac.il/consurf_index.php, [25, 26]). Lower negative values correspond to higher conservation. Positions are graded according to calculated scores 1-through-9, grade 1 being the lowest conservation and grade 9 the highest conservation.
**The multiple sequence alignment of Family I PPases generated by ConSurf and used for conservation score calculations included sequences sampled from various biological groups without filtering based on taxonomy or intracell location.

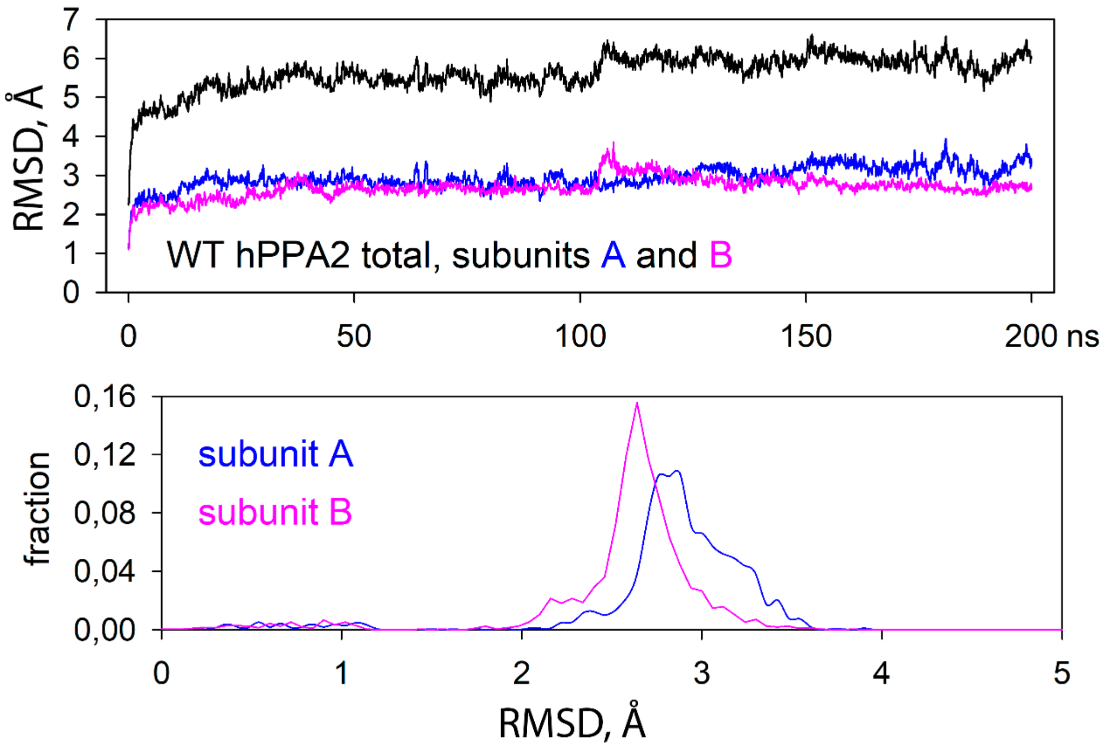

Figure S1. RMSD analysis of hPPA2. (top plot) RMSD of backbone atoms of a total protein (black line) and subunits A (blue line) and B (pink line) of dimeric hPPA2 from the initial structure over the entire simulation. (bottom plot) RMSD histograms for subunits A and B.

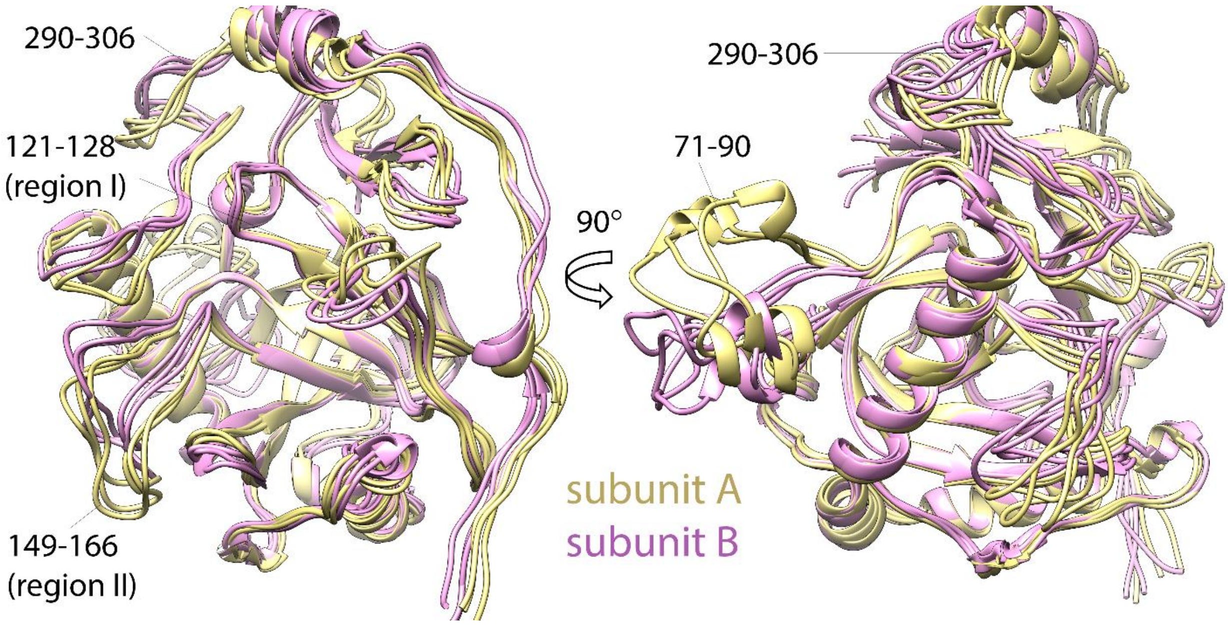

Figure S2. Superposition of subunits A (shown in yellow) and B (shown in pink) from several random frames of an MD trajectory after equilibration.

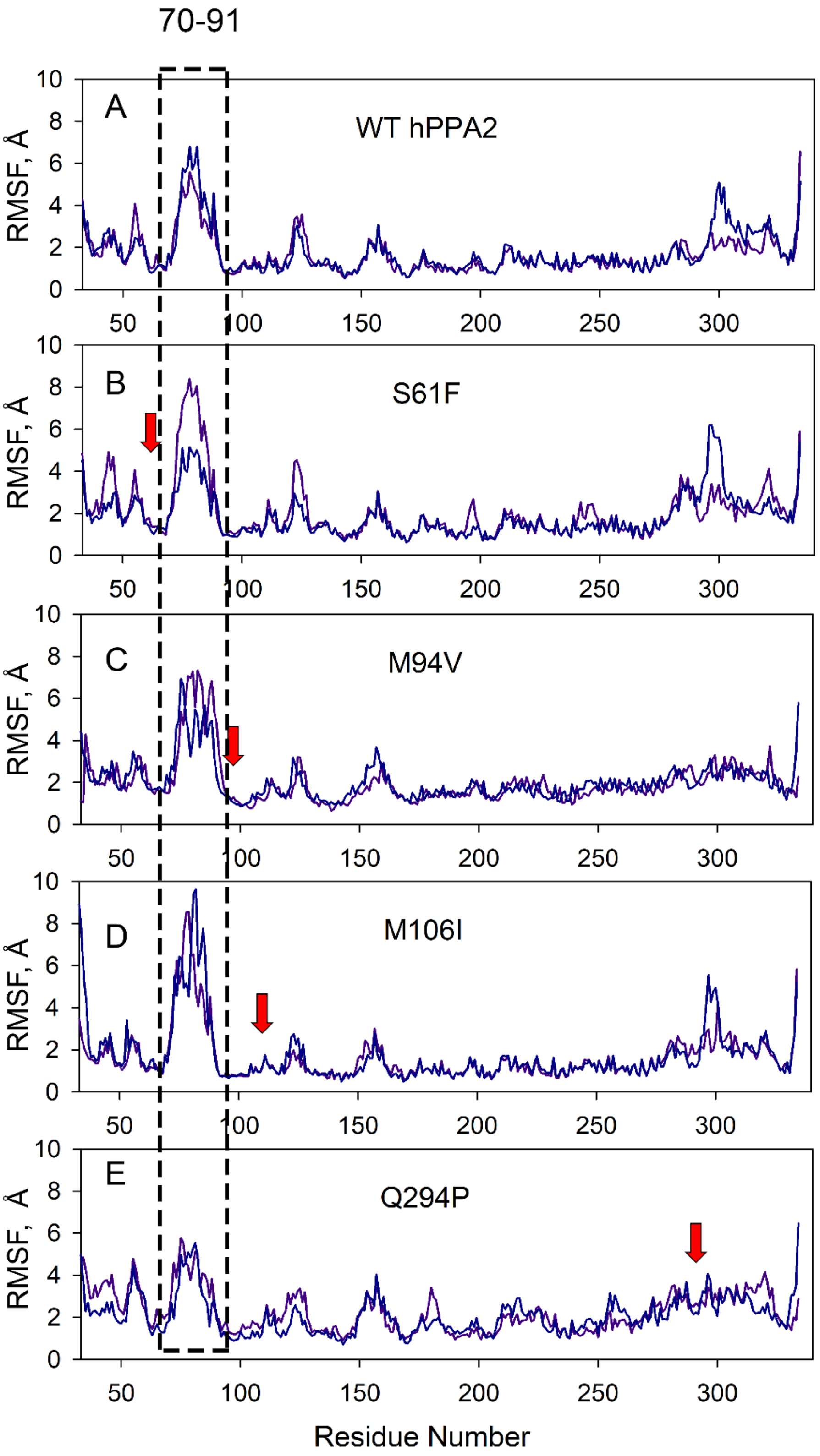

Figure S3. RMSF profile of hPPA2 (A) and its mutant variants S61F (B), M94V (C), M106I (D), and Q294P (E). The data for subunits A (blue lines) and B (purple lines) are shown for each protein. Sites of mutations are shown with the red arrows. Ω-loop 71-90 is boxed with the dashed line, flexible regions around the active sites are labelled as I-III.

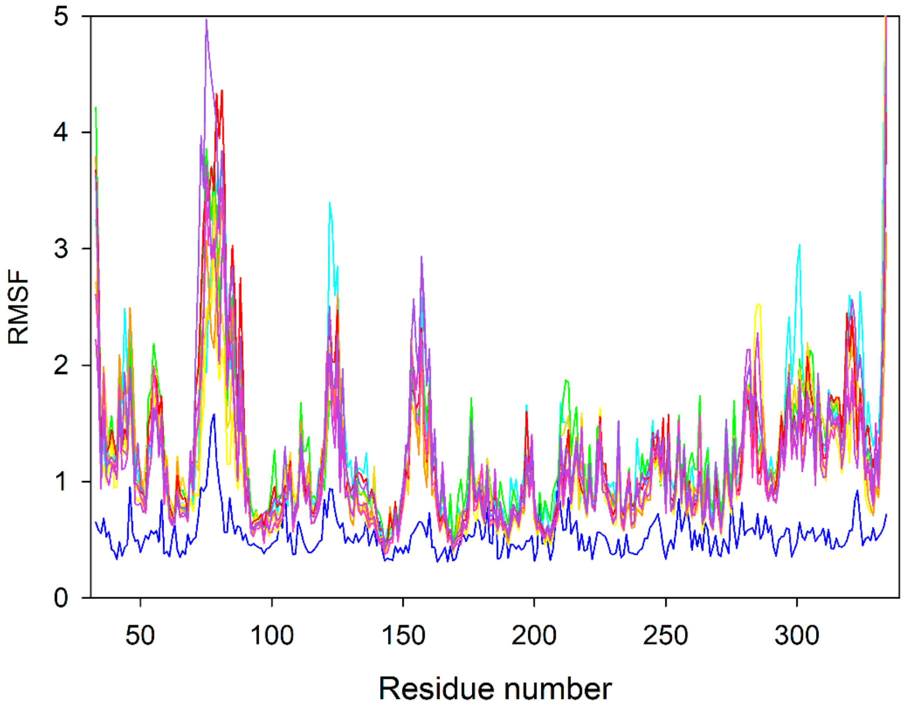

Figure S4. RMSF profile of subunit A of hPPA2 calculated for the different parts of a trajectory. The lines are colored by frame range using rainbow scale from blue (first 100 frames) to purple (last 100 frames).

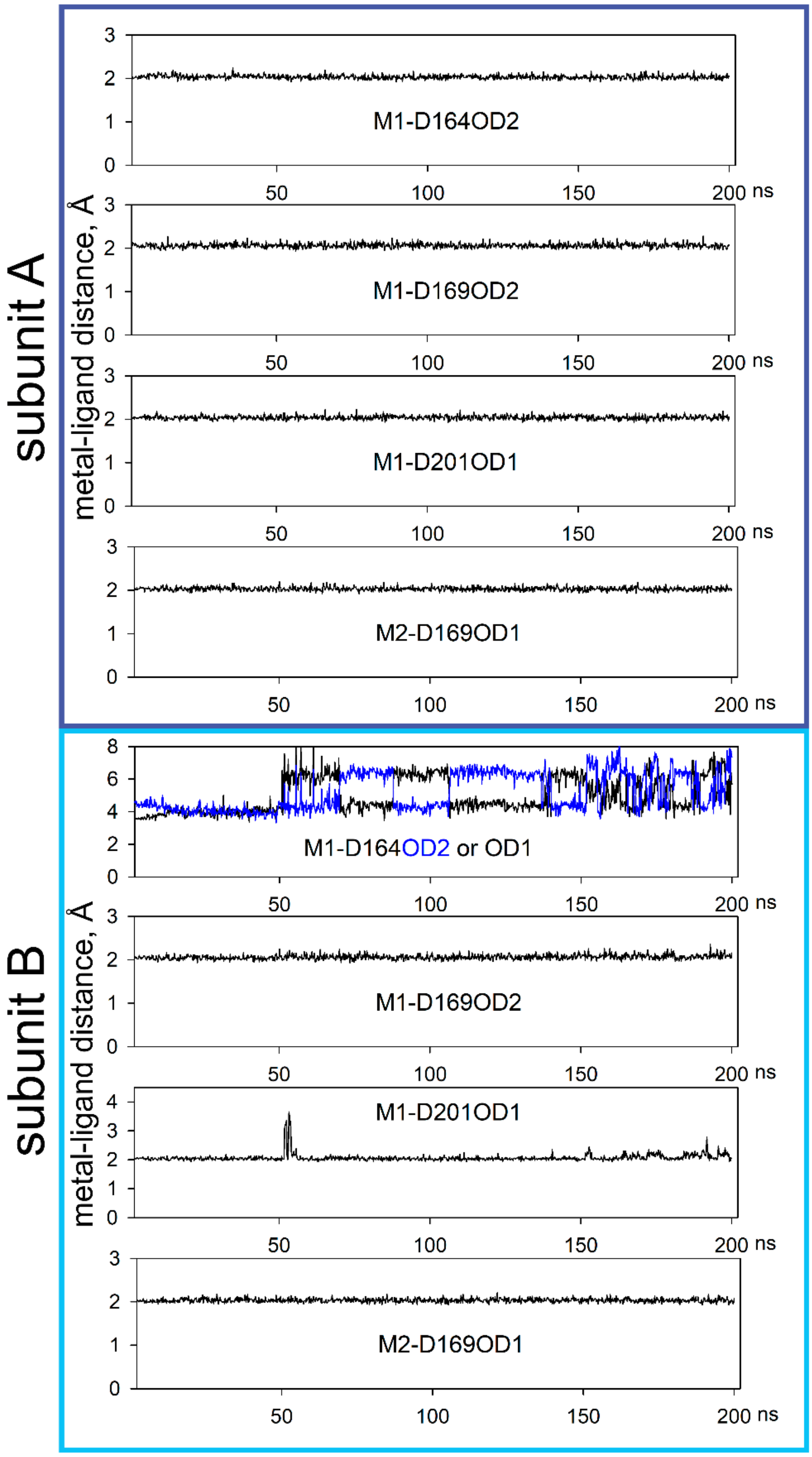

Figure S5. The distances from metal ions M1 and M2 in subunits A and B to their protein ligands.

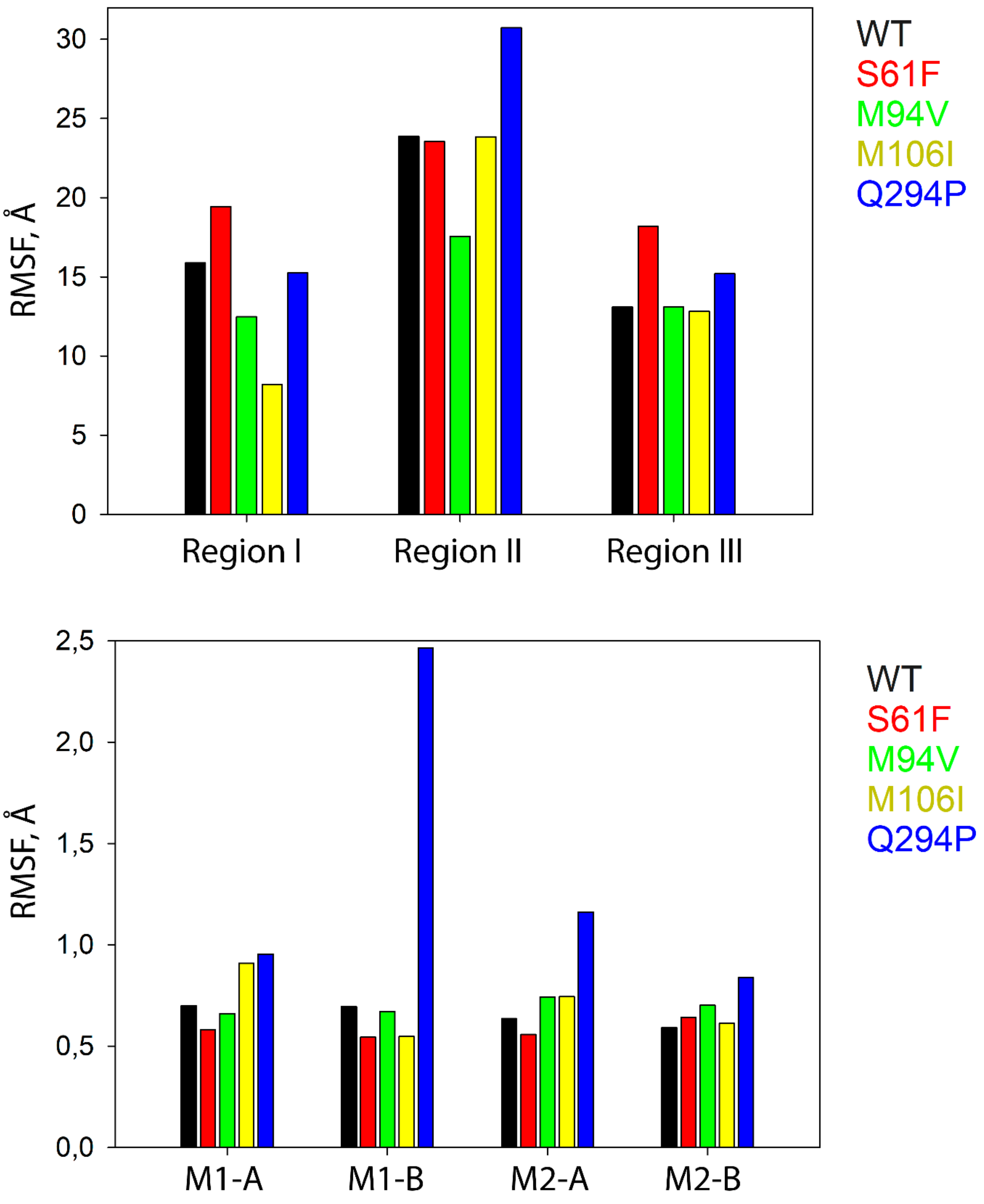

Figure S6. (A) RMSF of the regions I (residues 121-125),II (residues 152-164) and III (residues 189-201) in the course of simulation. (B) RMSF of the metal ions M1 and M2 in subunits A and B in the course of simulation. The values are given for the WT hPPA2 and the mutant variants.

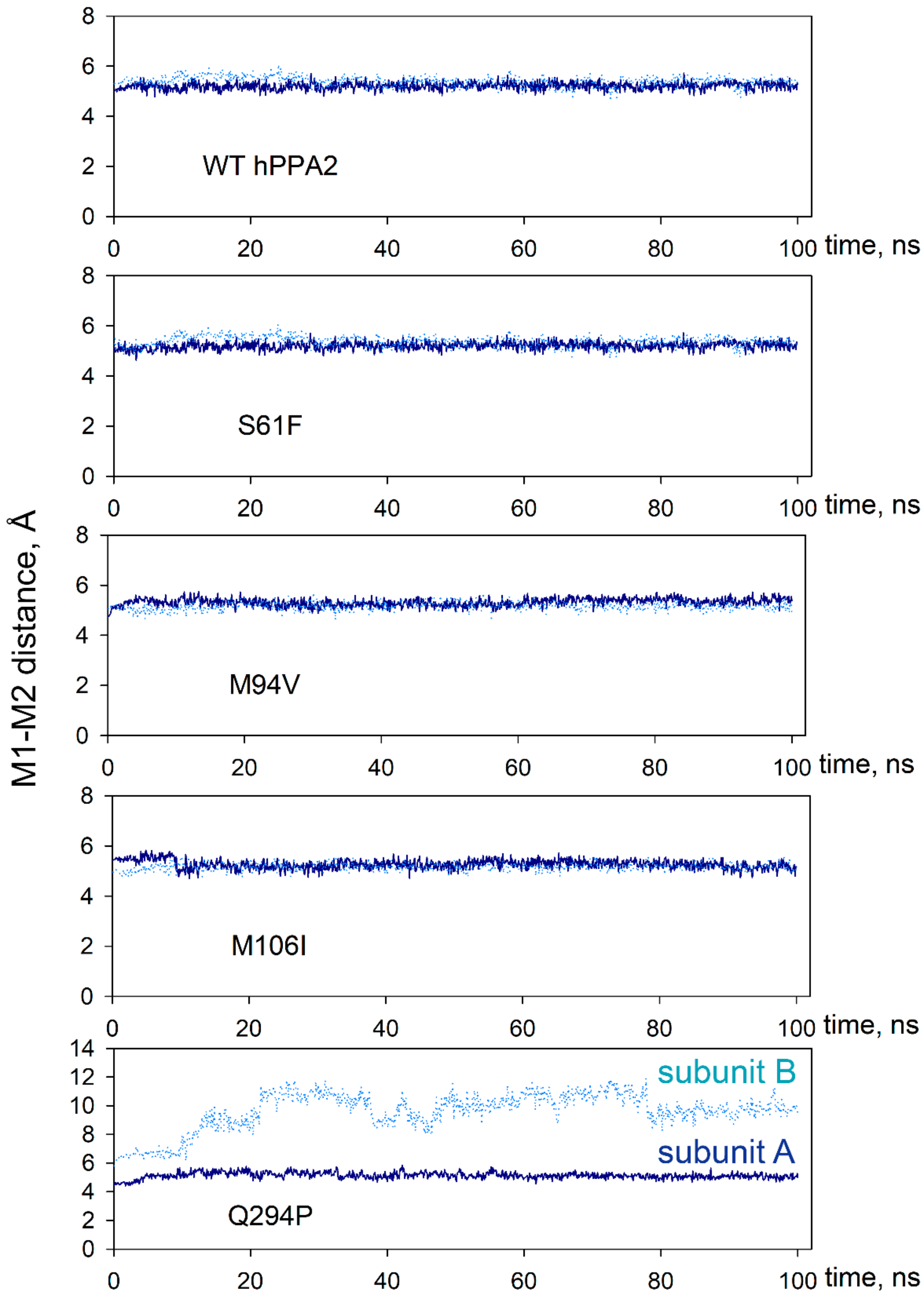

Figure S7. The distances between metal ions M1 and M2 in the WT hPPA2 and the mutant variants.

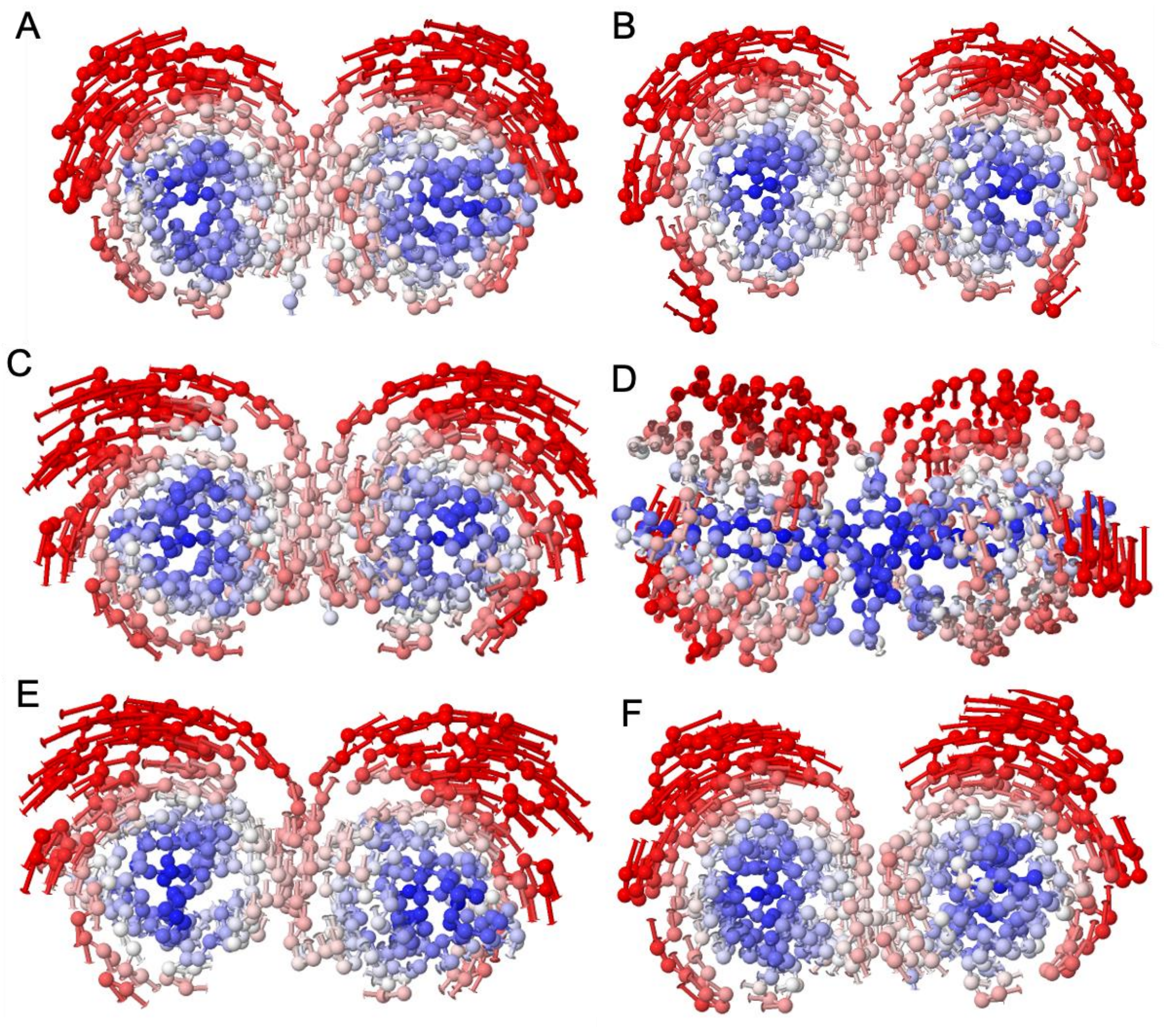

Figure S8. Mode 1 of the NMA analysis calculated for the hPPA2 structure at random frames of the MD simulation: (A) frame 50, (B) frame 150, (C) frame 200, (D) frame 600, (E) frame 1100, (F) average after equilibration. The pictures are copied from the DynOmics server (http://gnm.bahargroup.org/ [34]).

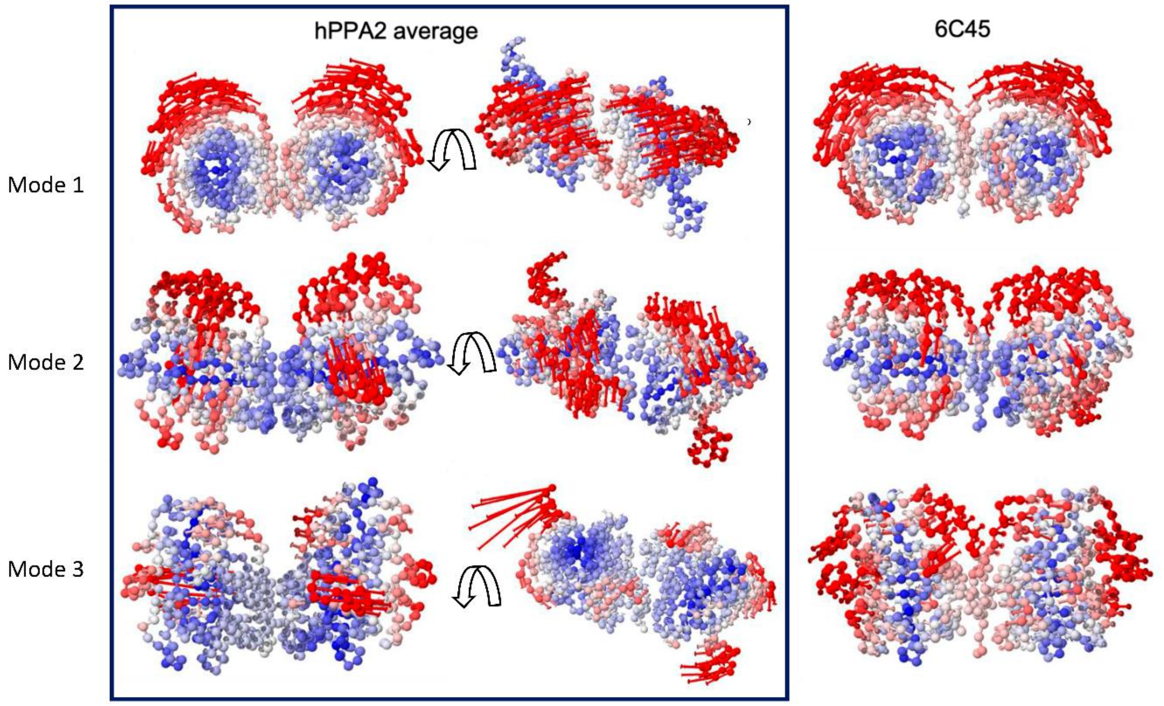

Figure S9. Modes 1-3 of the NMA analysis calculated for hPPA2 (average structure after equilibration) and hPPA1 (6c45 [31]). For hPPA2, front view and top view are shown; for hPPA1, front view is shown. The pictures are copied from the DynOmics server (http://gnm.bahargroup.org/ [34]).

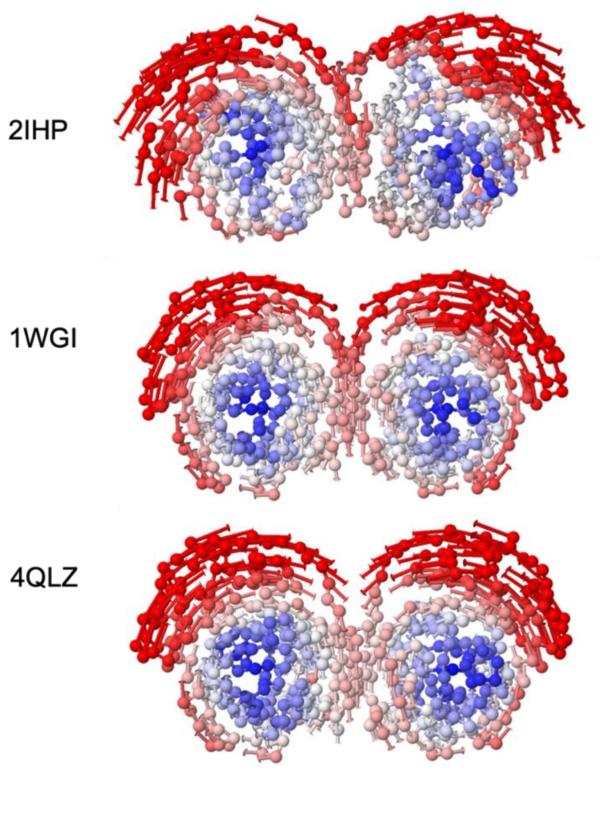

Figure S10. Mode 1 of the NMA analysis calculated for the crystal structures of different PPases: Sc-PPase (1WGI [30] and 2IHP [31]) and PPase from *Schistosoma japonicum* (4QLZ). The pictures are copied from the DynOmics server (http://gnm.bahargroup.org/ [34]).

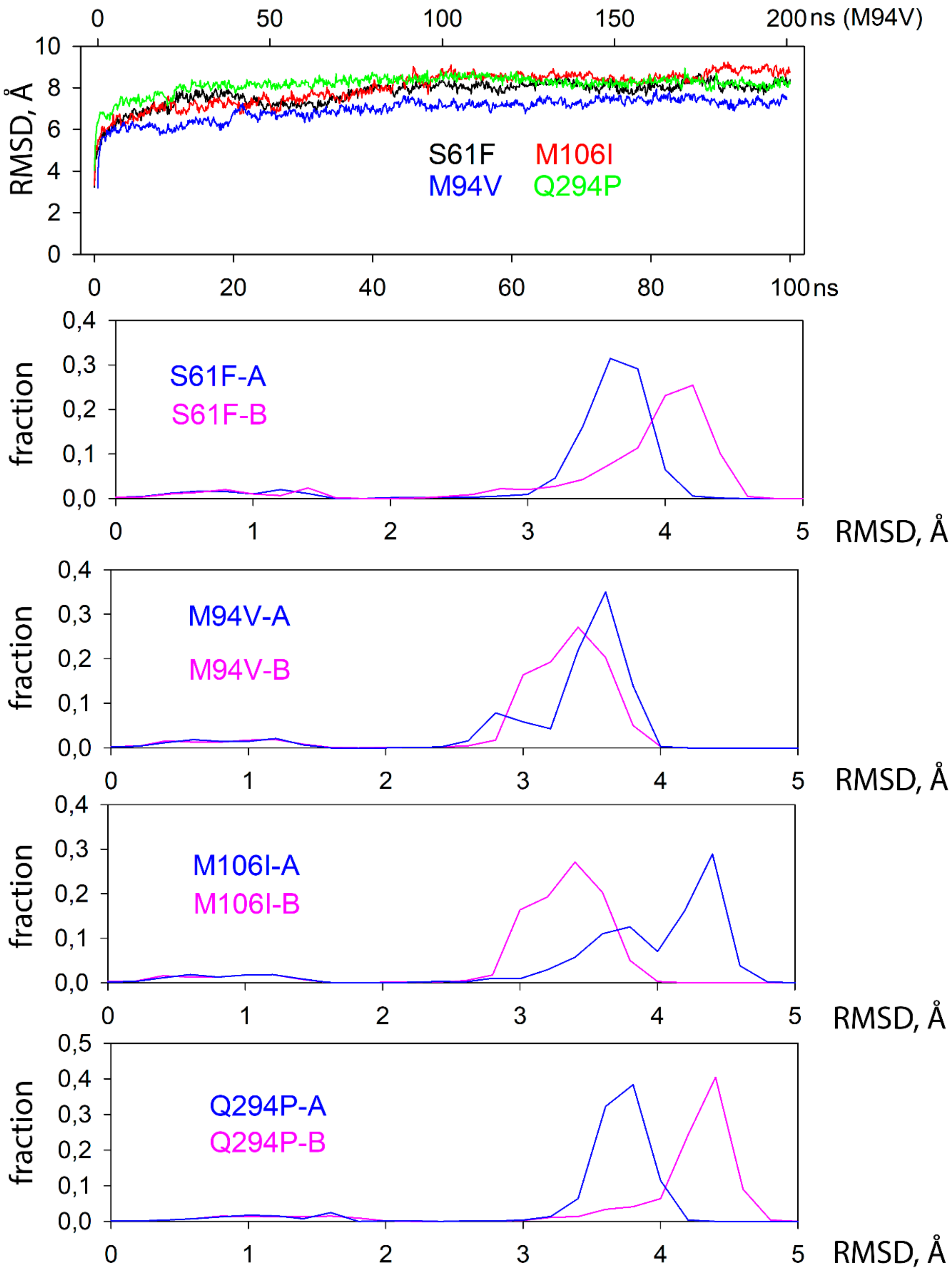

Figure S11. Total RMSD of dimeric molecules of the mutant variants of hPPA2 (top plot) and histograms of their distribution for the subunits A (blue lines) and B (pink lines).

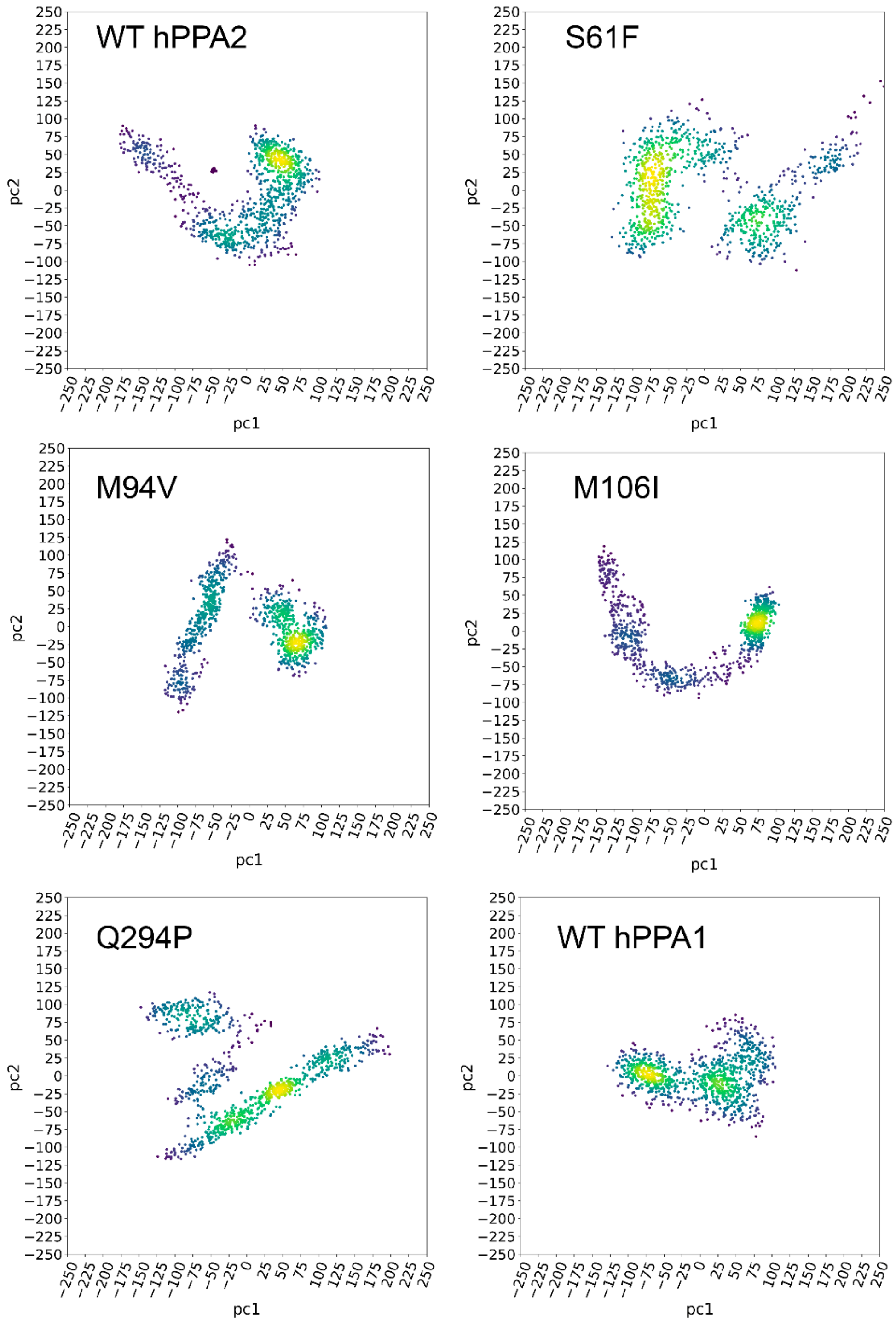

Figure S12. PCA analysis of the MD trajectory of mutant variants of hPPA2. Data are colored by population density using temperature scale from purple (lowest density) to yellow (highest density). The data for the WT hPPA2 and WT hPPA1 are given for comparison.

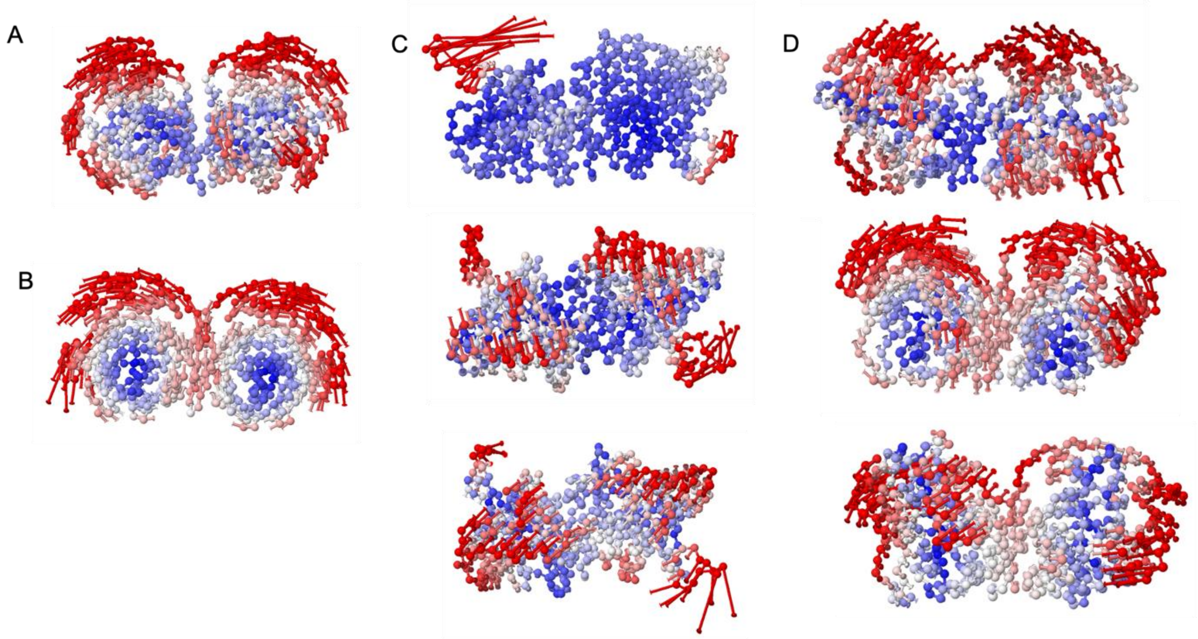

Figure S13. NMA analysis of mutant variants of hPPA2. (A) Mode 1 of Ser61Phe. (B) Mode 1 of Met94Val. (C, top to bottom) Modes 1-3 of Met106Ile, view from the top of the molecule. (D, top to bottom) Modes 1-3 of Gln294Pro. The pictures are copied from the DynOmics server (http://gnm.bahargroup.org/ [34]).
